# Simultaneity in the millisecond range as a requirement for effective shape recognition

**DOI:** 10.1186/1744-9081-2-38

**Published:** 2006-11-29

**Authors:** Ernest Greene

**Affiliations:** 1 Laboratory for Neurometric Research Department of Psychology University of Southern California Los Angeles, CA 90089-1061, USA; 2 Neuropsychology Foundation, Sun Valley, CA 91353, USA

## Abstract

Neurons of the visual system are capable of firing with millisecond precision, and synchrony of firing may provide a mechanism for "binding" stimulus elements in the image for purposes of recognition. While the neurophysiology is suggestive, there has been relatively little behavioral work to support the proposition that synchrony contributes to object recognition. The present experiments examined this issue by briefly flashing dots that were positioned at the outer boundary of namable objects, similar to silhouettes. Display of a given dot lasted only 0.1 ms, and temporal proximity of dot pairs, and among dot pairs, was varied as subjects were asked to name each object. In Exp 1, where the display of dots pairs was essentially simultaneous (0.2 ms to show both), there was a linear decline in recognition of the shapes as the interval between pairs increased from 0 ms to 6 ms. Compared with performance at 0 ms of delay, even the 2 ms interval between pairs produced a significant decrease in recognition. In Exp 2 the interval between pairs was constant at 3 ms, and the interval between pair members was varied. Here also a linear decline was observed as the interval between pair members increased from 0 ms to 1.5 ms, with the difference between 0 ms and 0.5 ms being significant. Thus minimal transient discrete cues can be integrated for purposes of shape recognition to the extent that they are synchronously displayed, and coincidence in the millisecond and even submillisecond range is needed for effective encoding of image data.

## Background

A cornerstone principle of neurophysiology is the idea that neurons are either intrinsically designed to be selective with respect to the stimuli to which they will respond, or through connections with other units, can be made to be selective [1-4].

A corollary is the concept of a "rate code," this being the notion that the strength or salience of the stimulus is reflected in the average rate at which the cell fires [5]. In this regard, it is assumed that the timing of individual spikes is random and must be averaged over some interval – generally thought to be in the 20–200 ms range.

This time interval seems consistent with various perceptual phenomena, such as the frequency at which one sees fusion of a flickering stimulus, that which provides for smooth motion in a rapid sequence of still images, and the duration of visible persistence resulting from a brief flash. The fact that an observer can combine partial shape cues over a hundred milliseconds or more to achieve object recognition also suggests that exact timing of the spike signal is not critical.

Eriksen and Collins [6,7] for example, examined the interval across which two dot patterns could be integrated to allow recognition of a three-consonant trigram. A portion of the dots needed to see the letters of the trigram were contained in each pattern, and random dots were added so that the letters could not be identified by inspection of either pattern alone. However, when presented in succession the information from the two patterns could be combined to allow successful recognition over an interval upward of 100 ms.

A prior study from this lab used a similar approach, i.e., the minimal transient discrete cue protocol [8], in which dots that marked the boundary of namable shapes were broken into two subsets. The number of dots in the subsets allowed for successful recognition with a 75% probability if both subsets were shown very briefly and with no delays. The ability to integrate the information from brief, successive display of the two subsets was a function of room illumination and of the time interval inserted between them. With dim illumination recognition levels fell only by half with a subset interval of 80 ms, and in the dark the hit rate fell less than 25% when the interval between the two subsets was 270 ms.

Results such as these show that shape cues can be combined over many tens or even hundreds of milliseconds. This suggests that the exact timing of spikes being sent forth from the retina is relatively unimportant for conveying shape cues. Put otherwise, and with specific reference to the recognition of shapes using briefly flashed dots, one would think that recognition should not be much affected by the order in which the dots were presented, or small differentials of time interval.

It is somewhat surprising, therefore, to learn that neurons can respond to stimuli with millisecond precision, and to hear proposals that synchrony of firing may be essential for image encoding and object recognition. Von der Malsburg [9,10] was among the first to suggest that coordinated firing of neurons might be used to specify what stimulus elements belong to a given object and to differentiate among the objects in a scene. This has been called the "binding hypothesis." One aspect of this hypothesis relates to the processing of extended contours that cross two or more non-overlapping receptive fields. Here it is proposed that synchronous firing provides a special signal that affirms the unity of the contour stimulus. In support of this possibility, coordinated firing across separate receptive fields in response to contours and gratings has now been reported for the retina as well as cortex [11-17].

It would be good to determine whether synchronous neural activity provides a special benefit for processing of cues needed for object recognition. This issue was tested in two experiments using stimulus conditions that would be expected to generate various degrees of synchronized neural response. Similar to the methods used in the earlier report [8], boundary dots were briefly displayed to elicit recognition of namable objects, mostly animals and manufactured items. The boundary dots were displayed in pairs, with various time intervals being inserted between successive pairs and/or between the pair members. The results indicate that simultaneity in the millisecond and even submillisecond range has a major influence on whether the stimuli can elicit recognition of the objects.

## Methods

Recognition judgments were collected from a total of 22 subjects, 8 for Exp 1 and 14 for Exp 2, using the minimal transient discrete cue (MTDC) protocol [8]. Except for the timing conditions detailed below, the stimuli to be judged and the task conditions were the same for both experiments.

Subjects were asked to name objects, each of which was suggested by a set of dots that marked locations at the boundary of the object, with the dots being displayed very briefly and in rapid succession. This will be described as display of a "shape pattern," or simply "shape" with the understanding that the pattern was designed to provide the minimal cues needed for naming the object.

One hundred fifty shape patterns, listed in Table 1, were shown to each subject. To create each shape pattern, an image of each object was sized and discretized so that the largest dimension of the object, either vertical or horizontal, fit to the edges of a 64 × 64 grid. Then a cursor was moved to trace the outer boundary of the object, marking the grid cells that were crossed by that boundary. This provided an x,y address for each marked cell, and the table of addresses provided the basis for subsequent display using a 64 × 64 array of LEDs. This LED array is hereafter described as the "display board."

**Table 1:** The names of shapes used in both experiments are listed, and for each shape, the table also provides the following information: Perimeter: the number of dots in the full inventory of boundary locations ; Area: the number of dots enclosed within the perimeter, and including the perimeter dots; Skip: the skip factor, which specified that every Nth dot would be included in the sample that was shown to a given subject; Dot% and Dot#: the percentage and number of dots that were displayed as a result of applying the skip factor.

Shape #	Shape Name	Perimeter	Area	Skip	Dot%	Dot#
1	alarm clock	276	2588	5	20.29	56
2	anchor	210	664	6	16.67	35
3	angel	240	1486	4	25	60
4	antique car	180	1565	4	25	45
5	antique chair	202	1329	3	33.66	68
6	baboon	316	1398	3	33.54	106
7	baby bottle	147	1228	3	33.33	49
8	badge	160	2060	3	33.75	54
9	banana	180	1385	8	12.78	23
10	bat	156	786	5	20.51	32
11	bear	213	1527	4	25.35	54
12	bee	309	1453	3	33.33	103
13	beetle	269	1345	3	33.46	90
14	bell	156	829	4	25	39
15	binoculars	176	1592	3	33.52	59
16	boot	183	1708	8	12.57	23
17	bottle	143	866	8	12.59	18
18	bowling pin	134	890	5	20.15	27
19	buffalo	238	1688	3	33.61	80
20	bull	302	1270	3	33.44	101
21	burro	359	1426	4	25.07	90
22	butterfly	306	2055	10	10.13	31
23	c clamp	267	952	3	33.33	89
24	camel	282	1956	7	14.54	41
25	candelabra	312	750	4	25	78
26	cap	158	1530	4	25.32	40
27	car	136	834	8	12.5	17
28	cat	248	1532	5	20.16	50
29	chair	217	1857	5	20.28	44
30	chick	177	1252	6	16.95	30
32	christmas tree	190	1423	3	33.68	64
33	coat	271	2365	3	33.58	91
34	coat hanger	160	649	6	16.88	27
31	cordless drill	240	1835	3	33.33	80
35	cow	256	1499	5	20.31	52
36	cowboy boot	189	1864	10	10.05	19
37	dagger	183	748	7	14.75	27
38	deer	353	1354	7	14.45	51
39	desk lamp	223	588	4	25.11	56
40	dinosaur	209	827	4	25.36	53
41	dog	280	1517	6	16.79	47
42	dragonfly	246	1068	3	33.33	82
43	duck	172	1025	6	16.86	29
44	dumbbell	185	1837	5	20	37
45	elephant	261	1761	6	16.86	44
46	fighter jet	240	1154	6	16.67	40
47	fire extinguisher	293	1995	3	33.45	98
48	fire hydrant	184	1145	3	33.7	62
49	fish	198	1364	3	33.33	66
50	flask	144	905	3	33.33	48
51	flower	222	2704	3	33.33	74
52	flying pheasant	229	1235	4	25.33	58
53	fox	245	961	3	33.47	82
54	frog	371	2078	4	25.07	93
55	giraffe	353	1137	8	12.75	45
56	glasses	215	649	5	20	43
57	glove	216	1165	3	33.33	72
58	goose	164	916	5	20.12	33
59	gramophone	230	1687	4	25.22	58
60	guitar	151	760	7	14.57	22
61	gun	171	968	4	25.15	43
62	hammer (ball peen)	156	416	3	33.33	52
63	hammer (claw)	168	506	5	20.24	34
64	hand shovel	144	540	3	33.33	48
65	hat	161	1496	6	16.77	27
66	heart	171	2685	9	11.11	19
67	helmet	161	1912	4	25.47	41
68	hen	218	1190	8	12.84	28
69	hippo	255	1984	3	33.33	85
70	horse	348	1588	5	20.11	70
71	horseshoe	273	1310	9	11.36	31
72	house	207	2647	4	25.12	52
73	humming bird	162	747	9	11.11	18
74	industrial hook	208	1431	4	25	52
75	iron	226	2039	3	33.63	76
76	jack rabbit	245	1686	12	8.57	21
77	kangaroo	246	860	5	20.33	50
78	knife	133	355	6	17.29	23
79	leaf	259	1294	7	14.29	37
80	light bulb	145	1461	7	14.48	21
81	lion	283	1334	5	20.14	57
82	lizard	242	976	6	16.94	41
83	macaw	158	726	4	25.32	40
84	man	249	888	4	25.3	63
85	man's shoe	157	1167	8	12.74	20
86	microscope	288	1003	3	33.33	96
87	monkey	256	892	3	33.59	86
88	moth	257	1642	7	14.4	37
89	motor scooter	228	1214	4	25	57
90	motorcycle	239	1355	7	14.64	35
91	mushroom	187	1504	5	20.32	38
92	music stand	193	592	5	20.21	39
93	ostrich	244	843	9	11.48	28
94	pan	151	1239	3	33.77	51
95	passenger plane	243	1038	6	16.87	41
96	pear	146	1174	7	14.38	21
97	pelican	248	1389	3	33.47	83
98	pepper	156	1068	3	33.33	52
99	piano	298	1844	5	20.13	60
100	pickup	154	790	5	20.13	31
101	pig	220	1357	6	16.82	37
102	pipe	151	503	7	14.57	22
103	pliers	224	517	4	25	56
104	porpoise	168	860	7	14.29	24
105	pot	177	1754	4	25.42	45
106	power boat	199	1262	5	20.1	40
107	propane torch	151	728	3	33.77	51
108	ram	392	1682	3	33.42	131
109	rat	192	785	4	25	48
110	rhino	187	1247	3	33.69	63
111	rifle	135	257	5	20	27
112	rooster	249	1453	6	16.87	42
113	sailboat	210	1008	3	33.33	70
114	saxophone	242	902	6	16.94	41
115	scissors	250	1186	5	20	50
116	sea gull	254	1132	3	33.46	85
117	sea horse	172	626	4	25	43
118	sea lion	202	1675	3	33.66	68
119	shark	185	831	7	14.59	27
120	sheep	232	1587	3	33.62	78
121	ship propeller	262	1665	6	16.79	44
122	shorts	192	2309	5	20.31	39
123	sickle	176	473	3	33.52	59
124	slipper	139	830	7	14.39	20
125	snail	176	989	3	33.52	59
126	snake	173	407	4	25.43	44
127	sock	144	823	6	16.67	24
128	spider	363	1112	3	33.33	121
129	spoon	134	416	4	25.37	34
130	spray bottle	180	1034	3	33.33	60
131	starfish	211	1301	9	11.37	24
132	submarine	147	769	4	25.17	37
133	swordfish	200	593	6	17	34
134	table	289	1357	7	14.53	42
135	table lamp	184	1187	5	20.11	37
136	teapot	185	1930	4	25.41	47
137	teddy bear	238	1571	3	33.61	80
138	telephone	200	2012	6	17	34
139	tiger	236	1031	4	25	59
140	toilet	225	2301	3	33.33	75
141	tractor	238	1864	3	33.61	80
142	trumpet	216	895	3	33.33	72
143	turtle	171	1100	5	20.47	35
144	umbrella	199	1764	6	17.09	34
145	vase	164	1562	6	17.07	28
146	violin	174	800	4	25.29	44
147	windmill	243	1330	4	25.1	61
148	wine glass	234	2091	5	20.09	47
149	wolf	267	1441	4	25.09	67
150	woman's shoe	162	874	6	16.67	27

The shape patterns were shown on the display board under the control of a Mac G4 computer and microprocessor slave. Each LED emitted with a peak wavelength of 660 nm, with a rise/fall time of 50–100 ns, and with a luminance of 10 Cd/m^2^. Background luminance, measured from the wall on which the display board was mounted, was 1 Cd/m^2^. Subjects viewed the display board from a distance of 3.5 m. At this distance the diameter of each LED subtended 4.9 arc' of visual angle, center-to-center spacing was 7.4 arc', and the span of the full array was 7.7 arc° in each direction.

**Figure 1:**
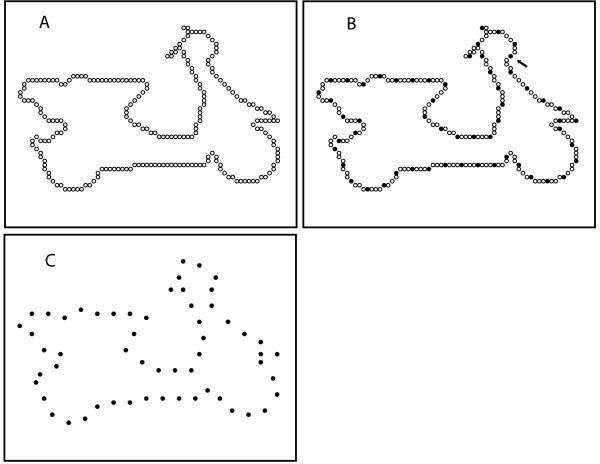
The average shape displayed 57 dots, this being every 4th dot from the full inventory of dots in the boundary. The full inventory of boundary dots for a shape that matches this average is shown in panel A. At B the method for choosing the display set is illustrated. To select this set for a given subject, a random dot was chosen as the starting point, here indicated by an arrow. Then, counting clockwise, every Nth dot was marked for inclusion in the set of dots to be displayed (every fourth dot for this example). The full complement of dots that would be shown, i.e., the display set, is provided in panel C.

Based on unpublish data gathered to formulate the protocols of the present and related experiments, the number of dots and their spacing was adjusted to provide approximate equivalence in potential for recognition of each shape pattern. Each shape was shown to a given subject only once, and only some of the dots in the boundary were shown. As illustrated in Fig. 1, selection of the display set for a given subject began by randomly choosing a starting point and then selecting every Nth dot, with N ranging from 3 to 10. Table 1 lists the number of boundary dots, the value of N, and the number of dots in the display set for each of the objects. This method of picking dots for the display set was the same for both experiments.

**Figure 2:**
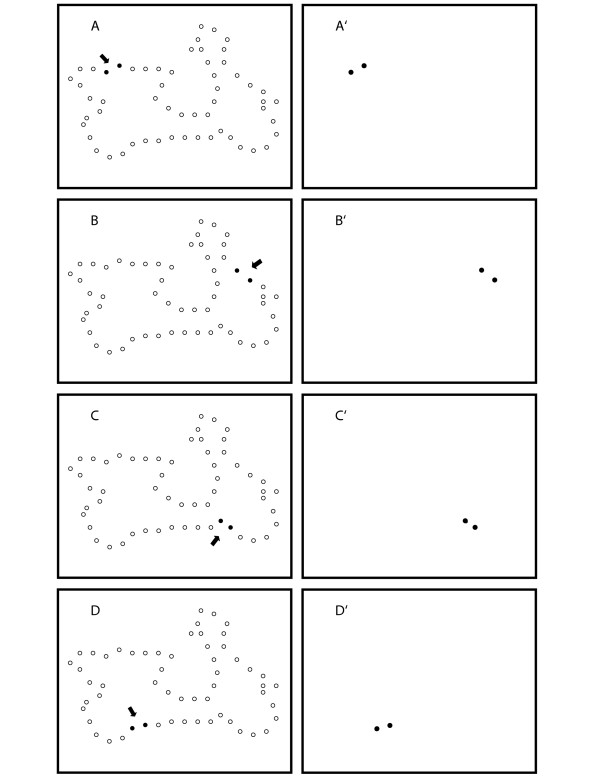
Adjacent dots from the display set were formed into pairs. The members of a given pair were shown sequentially, but the order of pair presentation was chosen at random. The left panels show the successive display of four pairs from the display set, and this sampling would continue until all pairs were shown. The right panels illustrate that the pairs would be seen as brief flashes of light at the specified positions within the array of LEDs. Dot size is not to scale for purposes of illustration.

For display of a shape to a given subject, adjacent dots of the display set were then yoked to form pairs. The two members of a given pair were always displayed sequentially, but the order in which pairs were displayed was random. Fig. 2 illustrates this protocol, with each of the panels on the left showing the full array of dots that constituted the display set for one of the objects, and the pairs (as filled circles) that were chosen for display. Note that the process would continue until all pairs were shown, with any odd remaining dot being displayed at the end of the sequence. Each panel on the right illustrates what would be displayed in Exp. 1 during a given 0.2 ms interval, with the time interval between successive pairs being varied, as detailed below.

**Figure 3:**
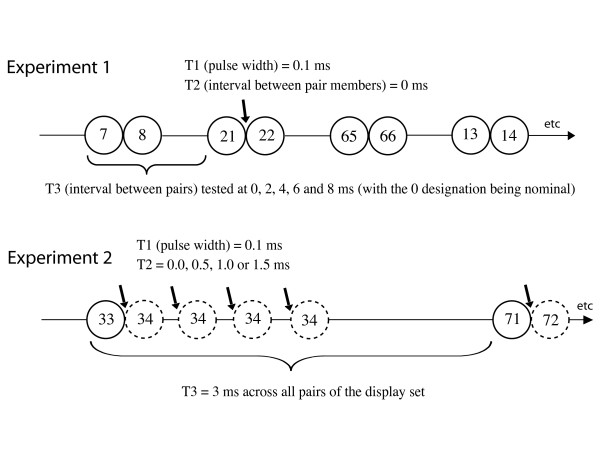
In Exp. 1, each dot of the display set was flashed for a duration of 0.1 ms, and there was no temporal separation between members of each pair. The temporal separation of pairs was varied from 0 to 8 ms. The time line has been expanded for the illustration of Exp. 2, most of it being used to illustrate the alternative intervals at which the second member of a given pair would be positioned. In this example, the pair formed by dots 33 and 34 are separated for an interval that varied between 0 and 1.5 ms, and the spacing between one pair and the successive pair (dots 71 and 72 in this illustration) was a constant 3 ms for all pairs in the display set.

Fig. 3 illustrates the timing conditions for the two experiments. All dots from the display set were shown one at a time, with pulse durations of 0.1 ms for each dot (designated as T1). The T2 interval specified time from offset of the first member of a pair till onset of the second member, and T3 specified the interval between successive pairs, measured from onset of the first pair till onset of the next pair. For Exp. 1 the T2 interval was a constant 0 ms., and there were five levels of T3, these being 0 (nominally), 2, 4, 6 and 8 ms. For Exp. 2 T3 was held constant at 3 ms, and there were four levels of T2, these being 0.0, 0.5, 1.0, and 1.5 ms. All timing was specified with a precision no less than 0.1 ms.

There was no demand for speed on the task, but subjects generally gave an immediate answer by saying the name, or indicating that no name for the shape pattern came to mind. The experimenter judged the acceptability of each answer without any information as to the timing level that had been displayed, and entered this data into a computer log.

## Results

The minimal transient discrete cue protocol is based on the concept that the information from the brief display of each dot must be combined across time for the full complement of cues to be sufficient for recognition. The question of interest is whether the degree of synchrony in the display of dot pairs (Exp 1) and/or of pair members (Exp 2) contributes to the integration progress

Subjects either could or could not identify a given shape, so the decision is binary. The appropriate model for such data is a generalized linear mixed model with binomial errors and a logit link function [18]. Logit values (log_e _(proportion/1 – proportion) were calculated, and treatment differences were compared using the standard error of the difference (SED) for these values. Subject and shape variables were treated as random effects in the analysis of data from each experiment, and T3 and T2 intervals were fixed effects for Exps. 1 and 2, respectively. A quadratic term was included in the model to test for possible nonlinear effects.

For the data from Exp. 1, using 8 subjects and judging the 150 shapes, there was a significant decline in recognition as a function of T3 interval (p < .001), with no indication of nonlinear effect (p = 0.75). A unit increase in separation of pairs corresponded to the odds of recognition being multiplied by a factor of 0.80 (95% confidence limit = 0.76, 0.83). There was a significant difference in hit rate at 2 ms, compared to performance at 0 ms (p < .05).

For Exp.2, with 14 subjects and again judging the 150 shapes, there was a linear decline in effect as a function of the T2 interval (between members of each pair) that was significant at p < .001. The unit increase in separation of pairs corresponded to the odds of recognition being multiplied by a factor of 0.35 (95% confidence limit = 0.19, 0.66). There was no indication of a nonlinear effect (p = 0.28). The difference between the recognition level at T2 = 0 versus at T2 = 0.5 ms was significant at p < .001. This was also found to be true when the data from just the first eight subjects was analyzed alone, so effect size for the two experiments is in the same range.

**Figure 4:**
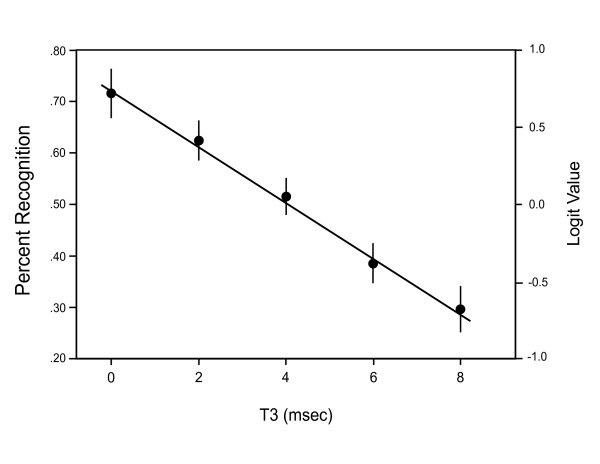
In Exp. 1, with 8 subjects and 150 shapes, each dot pair was presented within a 0.2 ms interval, and the T3 interval between pairs was varied. The hit rate declined across the 8 ms range for T3, and relative to hit rates at T3 = 0, the decline in recognition was significant even with a T3 of 2 ms. The right scale shows the logit values that were the basis for statistical analysis, and the error bars (+/- SEMs) should be interpreted against this scale. The ordinate on the left shows the corresponding levels of percent recognition.

**Figure 5:**
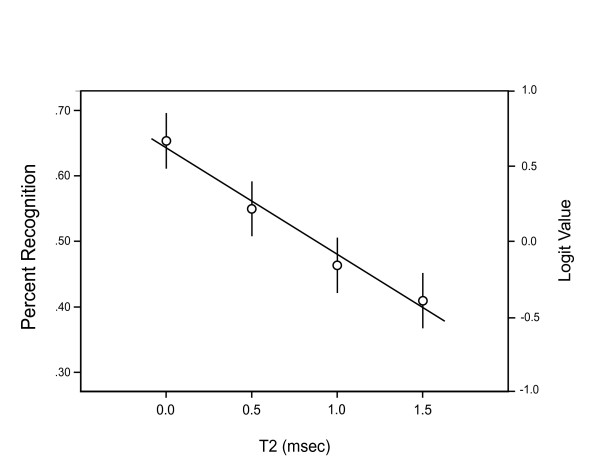
For Exp. 2 (14 subjects, 150 shapes), dot pairs were separated by a constant T3 interval of 3 ms, and T2 – the interval between members of each pair – was varied. Hit rates declined significantly with as little as 0.5 ms of separation between the pair members, and the decline in recognition was linear across the T2 intervals that were tested.

Mean levels of shape pattern recognition for the two experiments are illustrated in Figs. 4 and 5, along with regression lines that were calculated using only a linear model. Note that the right axis of each plot shows the logit scale that provides the appropriate measure of effect for the binary data, and error bars should be interpreted only in relation to this scale. The left scales show the means that were backtransformed into hit rates, these being almost identical to the means of the raw data.

Fig. 4 shows percentage recognition of the shapes to be over 70% when the dot pairs are displayed as rapidly as possible, i.e., with 0 ms of separation between offset of the last member of one pair and the onset of the first member of the next pair. Recognition of shapes declined as a linear function of T3, and as reported above, not only was the overall decline significant, even the drop from 0 to 2 ms proved to be significant. This may support an inference that synchrony in the millisecond range is a factor in "binding" of the shape cues, but see discussion of this issue below.

Fig. 5 shows recognition levels when 3 ms was provided between successive pairs, and with the temporal separation between the pair members being variable. The 3 ms T3 interval allowed for a hit rate in the 65% range when there was no temporal separation of the pair members, i.e., at T2 = 0. As T2 was increased the subject's ability to identify the shapes decreased, and as indicated in the analysis above, even a 0.5 ms interval between pair members produced a significant decline in recognition. This supports the proposition that neural responding is sensitive to submillisecond differentials in stimulus presentation, as discussed below.

## Discussion

As outlined at the outset, it is commonly thought that the timing of individual spikes is rather random, and that the essential information about stimulus attributes is conveyed by an average across intervals in the 20–200 ms range. A number of investigators have challenged that view [9-17,19-23], suggesting that precise synchronous firing of neurons provides a special designation of what stimulus components belong together. This has been described as "binding."

The binding concept is most often invoked to explain how one would define one shape from others that might be present in an image, though for the present work, it can be discussed in terms of aggregating partial cues for a single object. The goal is to combine those cue components that belong together. Von der Malsburg [9,10] argued that highly correlated activity, i.e., synchrony of firing across these shape components, is essential to that process. The degree of temporal contiguity would depend upon the specific linking to be done, but synchrony of spikes in the 1–10 ms range would be most likely needed in the early stages of image encoding.

The present work used very brief flashes from an array of LEDs to mark the outer boundary of shapes, and varied the timing of those flashes to determine how these manipulations affected recognition of the shapes. For Exp. 1, where zero separation of pulse-pairs was a constant condition, the average hit-rate was in the 70% range when there was no delay between successive pairs. Recognition dropped as the interval between pairs was increased, with hit rates being less than 30% when 8 ms was inserted between each pair. This falls within the time range that might be expected for initial image encoding [10], and these results might reflect a role for synchrony of cue components for eliciting recognition of the various shapes.

In evaluating whether the results of Exp. 1 relate to synchrony mechanisms, one must consider another hypothesis that is commonly invoked in discussions of iconic memory. It is widely believed that visible persistence provides the basis for integration, this being the sustained visibility of a brief stimulus for a period that is considerably longer than the stimulation itself [24-26]. This model specifies that recognition can be accomplished as long as one has not exceeded the duration of the integration window for essential cues. With an increase in the T3 interval, progressively more dots would exceed this duration, and one would expect hit rates to decline.

There are several reasons to reject an explanation that is based on the duration of visible persistence. First, a previous study from this laboratory [8] measured not only the time interval across which transient boundary dots could be integrated, but took independent measures of the duration of visible persistence. The duration of visible persistence of subjects did not predict the time interval across which the subjects could integrate the shape cues, nor did it predict the rate of decline that was observed as the interval between display subsets was increased.

Second, the incremental increase in the interval between dot pairs produces a total display time that is a multiple of the number of pairs. If the decline in recognition were due to exceeding the duration of the integration window, one would expect a geometric change in the rate of decline in recognition once the cumulative display time exceeded that interval. In the present experiment a linear decline was found, which seems more consistent with a synchrony hypothesis, which is further discussed below.

Third, the evidence suggests that the duration across which the transient discrete shape cues can be integrated is not a fixed interval. In the prior study mentioned above [8], the display set was broken into odd and even subsets, each containing half the dots to be shown. With dim room illumination, hit rates were in the 75–80% range when the interval between subsets was 20 ms or less, and they declined to about half that level with an interval of 80 ms. From the plot shown in Fig. 4, one can infer a comparable level of decline, i.e., a 50% drop in recognition levels from the maximum, when the interval between pairs was 6–8 ms. This provides a total display time, on average, of somewhere between 170 and 230 ms. This is over twice as long as the duration over which the odd-even subsets could elicit a 50% decline in recognition when subjects were tested with the room dim [8].

It is more plausible that the ability to combine successive dot pairs into a code that can effectively elicit recognition depends on the temporal contiguity between successive pairs. Based on the data from Exp. 1, the linkage that takes place, i.e., binding, appears to be a linear function of time intervals in the millisecond range.

The second experiment provides additional evidence that simultaneity contributes to the processing and integration of shape information. Separation of pulse-pairs was held constant at 3 ms, so total time to show all the dots was the same for each of the T2 intervals that separated the pair members. For the T2 = 0 condition, wherein stimulus pairs were virtually simultaneous, recognition levels were in the 70% range. Providing even 0.5 ms of separation between the pulses produced a significant drop in recognition, and with an interval of 1.5 ms the hit-rates had dropped into the 40% range. Thus simultaneous (or near simultaneous) presentation has a substantial benefit for integration of the successive stream of partial shape cues.

The benefit from millisecond and submillisecond simultaneity of stimulus pulses may reflect encoding operations taking place at the earliest stages of visual processing, i.e., in the retina. Full field stimulation of ganglion cells with random flicker causes a reduction of spontaneous activity, and these neurons then provide only sparse firing that is tightly linked to the brightness transitions [27-33].

Meister et al. [32] as well as Brivanlou et al. [34] examined the stimuli that would elicit correlated activity of neighboring retinal ganglion cells, and concluded that the synchrony was provided by joint activation of overlapping portions of their receptive fields. They suggested that spiking amacrine cells provide the basis for the synchronous firing.

Mastronarde [35] found synchrony in On and Off Y cells, wherein the mutual influence was restricted to cells of the same class. Antidromic activation of one of these cells would lead to an increase in the firing rate of neighbors that begin in about 0.5 ms and lasted for 1.5 ms. This investigator suggested that the joint activity was from direct electrical coupling by gap junctions rather than being a response to common input. Gap junctions would provide electrotonic linkage between adjacent neurons, essentially combining their receptive fields.

Several groups have found synchronized firing to a moving slit from retinal ganglion cells [16,17,36]. At least for direction-selective On cells of rabbit, chemical blockade of gap-junction communication abolished synchronous firing, possibly by disrupting input from wide-field amacrine cells [36].

DeVries [37] suggested that gap junctions were responsible for yoking the responses in 4 of the 5 classes of ganglion cells where synchrony was observed, and similar results were reported by Hu and Bloomfield [38] for Off-center ganglion cells of rabbits, but not for On-center cells. Hidaka et al. [39] examining rat retina using dual patch recordings and tracer labels, and demonstrated dendrodendritic gap junctions and electrotonic coupling of alpha ganglion cells of the same type, including On-center cells.

Nirenberg et al. [40] argue against the hypothesis that correlated firing provides a special signal. Using an information measure, they examined the firing patterns of isolated mouse retina, and conclude that 90% of the information that can be recovered from the cell firing can be derived from the independent activity of the separate ganglion cells responses. It can be said, however, that the final 10% that could not be accounted for may well be highly significant information, and in particular may signal the position of key boundary markers that allow the object to be identified.

Roelfsema et al. [41] and Palanca and DeAngelis [42] found little evidence that synchrony serves to bind contours that were part of a common form. Their results challenge at least the most general form of the synchrony/binding hypothesis [10,22], but cannot be taken as evidence that synchrony provides no perceptual benefit. Even if synchrony does not serve to bind all contours, it might provide a means to mark events that are temporally coincident, such as a common moving edge.

Although synchrony-based encoding may begin in the retina, it is possible that the process continues, yielding correlated firing of cortical neurons. For the present results, an increase in the interval between pair members (T2) produced a 17% decline in recognition for each millisecond that was added to the interval, whereas recognition declined by only 5.3% for each millisecond that was added between pairs (T3). This suggests two separate processing stages, retinal and cortical, with the former being especially sensitive to the time interval.

It is certainly the case that cortical neurons are capable of synchronous firing, and most of the evidence and theorizing about the role of synchrony for binding of stimulus attributes is based on recordings taken from cortex. [For reviews, see [23], [43-46]] In this regard, the present results lead to a slight modification of the proposal that synchrony contributes to the analysis of boundaries, in that isolated dots were used rather than contours. There can be no doubt that these boundary markers provide the necessary information for shape recognition, for the shapes are indeed identified. So we can say that simultaneity in the presentation of boundary markers, and most likely the synchronized neural responses that they generate, contributes to the binding of information that is important for ultimate recognition of shapes.

## Conclusion

Objects can be identified from brief display of dots that mark the outer boundary of the objects. Recognition drops as a linear function of temporal separation in the display of successive dot-pairs, or with temporal separation of members of a pair. In the latter condition, recognition is significantly impaired if as little as half a millisecond of time is provided between offset of first and onset of the second member of the pair. These results support proposals based on neurophysiological findings that argue for synchrony of firing as a special encoding process.

## Abbreviations

arc° - degrees of visual angle

arc' - minutes of visual angle

Cd/m^2 - ^candela per meter squared

GaAlAs - gallium, aluminum and arsenic

LED - light emitting diode

Log_e - _natural log

m - meters

ms - milliseconds

N - number used to specify which dots from address list will be displayed

nm - nanometers

ns - nanoseconds

p - probability

T1 - pulse width

T2 - temporal separation between members of subset pairs

T3 - temporal separation between subset pairs

## Competing interest statement

The author(s) declares that he has no competing interests.

## Acknowledgements

Computer programming for conduct of this research was done by David Gorin, DarkHorse Software. LED emission was measured by Dr. Andrew Jones, USC Space Science Center. Statistical analysis was done by Leigh Callinan, Bendigo Scientific Data Analysts. This research was supported by the Quest for Truth Foundation.
